# Use of Artificial Intelligence in the choice of sperm for ICSI - A Literature Review

**DOI:** 10.5935/1518-0557.20260008

**Published:** 2026

**Authors:** Lorrany Lima Ricarte, Raissa de Vasconcelos Cavalcanti, Paula Bruno Monteiro

**Affiliations:** 1 Centro Universitário Christus (UNICHRISTUS), Fortaleza, Ceará, Brazil; 2 Clínica Conceptus, Fortaleza, Ceará, Brazil

**Keywords:** human reproduction, neural networks, infertility, deep learning

## Abstract

This study aimed to verify, through a systematic literature review, the effects of using artificial intelligence (AI) for sperm selection in intracytoplasmic sperm injection (ICSI). Searches were conducted in the PubMed, SciELO and MEDLINE (Academic) databases, including full-text cohort studies, cross-sectional studies and clinical trials published between 2015 and April 2025 in English, Portuguese and Spanish. Animal studies, review articles, letters to the editor, case reports, duplicate records, incomplete papers and unavailable articles were excluded. Extracted data comprised author, year of publication, AI type, assessed stage/section and classification. Overall, the available evidence indicates that AI-based approaches may be effective for selecting sperm for ICSI.

## INTRODUCTION

According to the World Health Organization (WHO, 2023), infertility is defined as the inability of a couple to conceive a pregnancy after 12 months or more of regular unprotected sexual intercourse, which affects around 17.5% of the adult population. The male factor is responsible for around 30-50% of cases. Many couples therefore seek the help of assisted human reproduction techniques, with intracytoplasmic sperm injection (ICSI) being the most common in cases of male infertility (Eisenberg *et al.*, 2023).

For this purpose, there are several sperm capacitation techniques that select a group of sperm, including: swim up (SU) and density gradient centrifugation (DGC). Swim up is a technique known as the most basic method of sperm preparation. The principle is based on the speed of directional progression of sperm. Density gradient centrifugation is a technique in which sperm are placed on a continuous or discontinuous density gradient and then centrifuged. Separation occurs according to density and motility; the fastest sperm will migrate to the bottom of the tube (Baldini *et al.*, 2021). Subsequently, the capacitated sperm will be selected based on the evaluation of some seminal parameters, such as motility and morphology, and used in assisted reproduction techniques, such as ICSI. In this technique, embryologists select a single sperm cell based on its morphology and motility to inject into a single oocyte. However, these professionals face difficulties in selecting one sperm among millions, since gametes are fragile cells. Selecting just one cell involves a lot of responsibility on the part of the laboratory team, in order to avoid financial and emotional stress on patients (Asada, 2024).

With the increase in infertility cases and advances in assisted reproduction techniques, artificial intelligence (AI) has become an attractive tool for improving success rates, optimizing time and avoiding errors (Korfiatis *et al.*, 2023). Due to its speed and ability to analyze complex algorithms, AI has been used in a variety of areas, especially in the health sector, such as diagnosing and treating various diseases (Elias *et al.*, 2023) and improving the accuracy and efficiency of workflows (Kaul *et al.*, 2020). In reproductive medicine, AI began in the 20th century and is now an ally of embryologists (Medenica *et al.*, 2022).

Studies have shown that AI can provide more objective and accurate embryo quality assessments than trained embryologists. According to a study conducted in 2023, AI models had a median accuracy of 77.8% (range 68-90%) in predicting clinical pregnancy by using the patient’s clinical treatment information, compared to 64% (range 58-76%) when performed by embryologists. When imaging/time-lapse inputs and clinical information were combined, the median accuracy by AI models was higher, at 81.5% (range 67-98%), while clinical embryologists had a median accuracy of 51% (range 43-59%) (Salih *et al.*, 2023). In the same year, another study showed that embryo selection using an AI algorithm based on automatically measured blastocyst size could serve to improve the clinical outcome related to increased implantation potential. It was seen that such automation would increase the consistency and accuracy of blastocyst measurements (Fruchter-Goldmeier *et al.*, 2023).

Bori *et al.* (2022) used the KIDScoreD5 algorithm to identify embryos with a healthy chromosomal pattern and a high chance of resulting in a viable birth. The algorithm’s methodology consists of assigning categories to embryos based on criteria such as cleavage time and blastocyst appearance. By retrospectively analyzing 22,461 embryos, the researchers observed that those with a higher classification had significantly higher implantation and viable birth rates. These results highlight the effectiveness of KIDScoreD5 in differentiating embryos with similar morphology, identifying those with the greatest potential for development, which can help embryologists in clinical decisions.

Therefore, given some of the most significant applications of AI in fertilization techniques, it is clear that AI has the ability to minimize variability between operators and improve fertilization results (Fitz *et al.*, 2021). However, artificial intelligence has been extensively studied in embryo classification, but studies on the use of AI in sperm selection for ICSI are lacking. This study was therefore designed to relate the use of artificial intelligence to sperm selection.

## MATERIALS AND METHODS

### Type of study

This study is a systematic literature review. To this end, the *Preferred Reporting Items for Systematic Reviews and Meta-Analyses* (PRISMA) guidelines were used.

### Search strategy

To carry out the searches in this study, descriptors were initially checked using the Medical Subjects Headings (MESH). After selection, these descriptors were allocated in combination with a Boolean operator in a single search: *Neural Networks AND Sperm.* This search strategy was carried out in three different databases: Pubmed, Scielo and MEDLINE-Academic.

### Study eligibility criteria

To select the studies, the following eligibility criteria were adopted: clinical trials, cohort studies and cross-sectional studies that addressed the topic proposed by the authors. The studies evaluated in this review were published between 2015 and April 2025 in English, Portuguese and Spanish.

### Exclusion criteria

Animal studies; review articles; letters to the editor; case reports; articles with duplicates; incomplete articles and unavailable articles.

### Data collection

After the searches were carried out, the articles were evaluated according to their title and abstract and those that did not comply with the aforementioned criteria were excluded.

### Data extraction

Immediately after collection, these articles were read to obtain the following data: author’s name; year of publication; type of artificial intelligence; section evaluated and classification.

## RESULTS

**Figure 1:**
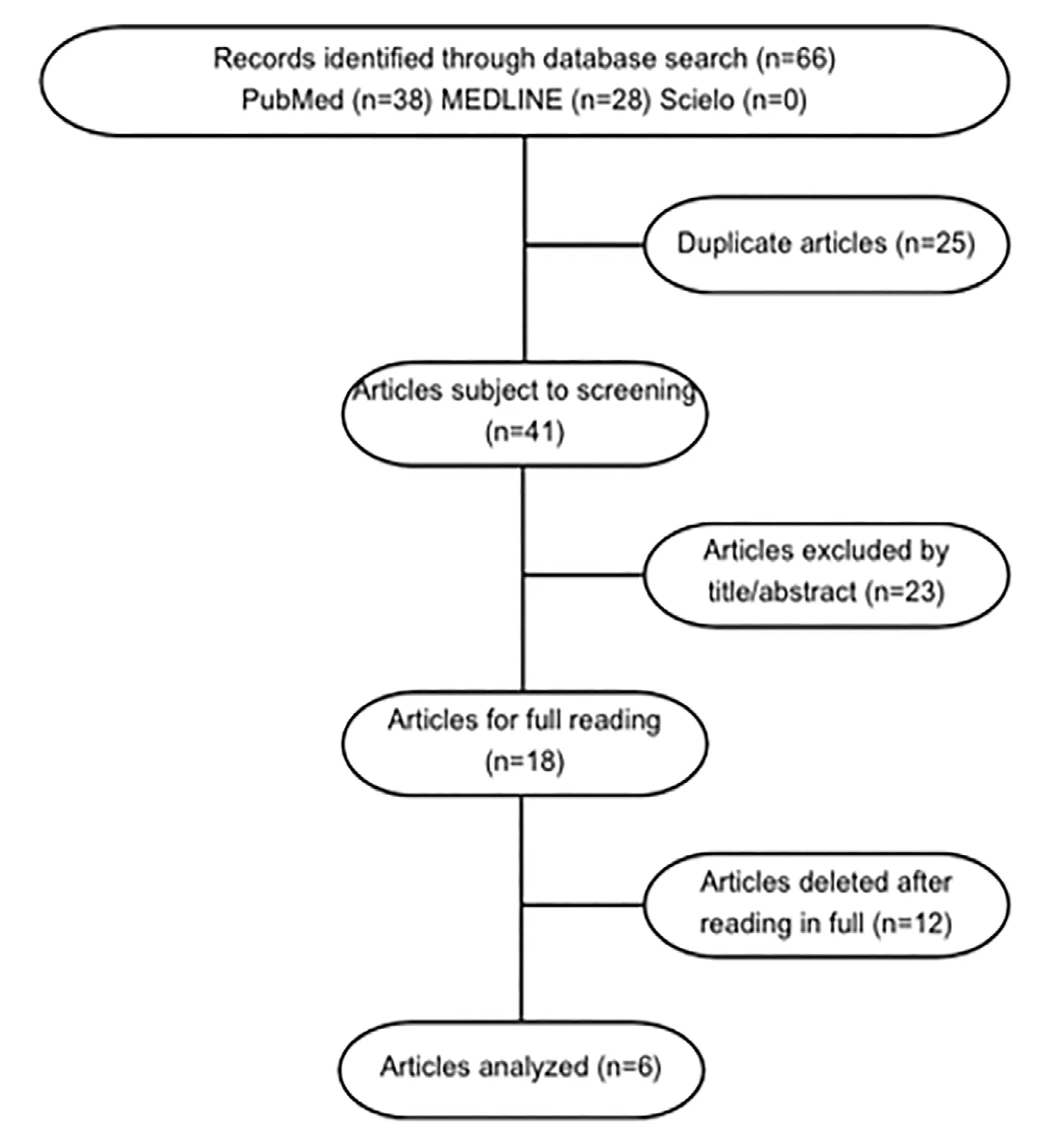
Methodological screening.

The records identified in the data search were: PubMEd (n=38), SciELO (n=0), MEDLINE - Academic (n=28), resulting in a total of 66 articles. After the initial search, 25 duplicate articles were removed, leaving 41 articles for screening. After reading the title and abstract, 23 articles that did not meet the inclusion criteria were excluded, with 15 articles excluded because they were reviews and 8 because they were studies with animals, leaving 18 articles for reading in full. After reading in full, 12 articles were excluded because they did not present the desired results of the review, and the other 6 were analyzed (Figure 1).

Of the 6 articles analyzed, three assessed morphology, two analyzed DNA fragmentation and one assessed motility. All the articles reported artificial intelligence as an important tool in selecting sperm for intracytoplasmic sperm injection. However, no study observed fertilization rates, blastocyst formation rates or pregnancy rates (Table 1).

**Table 1:** Artificial intelligence systems for sperm selection.

Author/ Year of publication	Type of artificial intelligence	Section evaluated	Classification
Kanakasabapathy *et al*. (2021)	MD-net (Xception-based)	Morphological	Normal or abnormal
Mirsky *et al*. (2017)	SVM (Support Vector Machine)	Morphological	Good or bad
Yang *et al*. (2024)	BlendMask	Morphological	Normal or abnormal
Noy *et al*. (2023)	MobileNet	DNA fragmentation	DNA quality
Noy *et al*. (2023)	CNN	DNA fragmentation	DNA quality
Haugen *et al*. (2023)	DCNN	Motility	Mobile or immobile

## DISCUSSION

### Sperm morphology

Currently, sperm morphology serves as the main selection metric in assisted reproduction practices (Björndahl *et al.*, 2022). In addition, Teves & Roldan (2022) point out that sperm morphology is directly associated with their function. Favorable characteristics of sperm morphology include a smooth, oval head, acrosome covering 40%-70% of the head, absence of large or multiple small vacuoles, slenderness of the midpiece and equal length compared to the head, residual cytoplasm up to one third the size of the head shape and texture of the head surface, percentage of acrosome area, presence or absence of vacuoles (Björndahl *et al.*, 2022). Furthermore, assessing sperm morphology is extremely important for diagnosing infertility and for possible treatments, as well as for choosing the assisted reproduction technique (Gatimel *et al.*, 2017).

Of the 6 articles found, 3 used AI algorithms to select sperm for morphology, as shown in Table 1. The study by Kanakasabapathy *et al.* (2021) used MD-net (based on Xception) trained with sperm images collected by the Proficiency Testing Service (PTS) of the American Association of Bioanalysts (AAB). And the selected sperm images were then classified as normal or abnormal based on morphology, using MD-Net trained on 2,899 annotated images. In the tests, the network achieved 86.99% accuracy (95% CI=83.37-90.07%) in morphological classification (n=415).

The study carried out by Mirsky *et al.* (2017) used images captured from more than 1,400 human spermatozoa from 8 different individuals, using phase interference microscopy (IPM). A computational method was developed to digitally extract the heads of male gametes from three-dimensional quantitative phase data, considering both the cell shape and its internal structure in 3D, as well as acquiring characteristics that describe the morphology of the sperm head. Subsequently, a selected group of these parameters was applied to train an artificial intelligence system based on Support Vector Machine (SVM). The aim was to automatically distinguish between properly shaped sperm and those with morphological anomalies. The classification model demonstrated high performance, achieving an area under the receiver operating characteristic curve of 88.59% and an area under the precision-recall curve of 88.67%, as well as accuracies of 90% or more.

Another study used BlendMask which is a sperm morphology analysis method that accurately segments and extracts data to obtain the head, midpiece and mainpiece comments and then uses the deep learning classification algorithm to classify and evaluate each part of the segmented sperm according to WHO standards. The algorithm accurately classified normal sperm and provided several explanations for abnormalities. Morphological accuracy was verified by multicenter clinical verification to be > 90%, consistent with the standard for clinical detection of sperm morphology and demonstrating high clinical application value (Yang *et al.*, 2024).

These studies corroborate the study by Thirumalaraju *et al.* (2019) which showed that the analysis of human sperm morphology using smartphone microscopy and deep learning was able to correctly identify samples based on morphological quality with 88.5% accuracy and a 95% confidence interval (CI) which proved that the (AI) algorithm can effectively separate samples with normal and abnormal morphological quality. In addition, artificial intelligence is capable of classifying healthy, oxidation-stressed, cryopreserved and ethanol-affected sperm, where ResNet-101 was used and provided an accuracy of 85.6%, in the study by Butola *et al.* (2020). Therefore, although artificial intelligence is highly desirable for detecting alterations and, consequently, for selecting sperm for the ICSI procedure, which can be used to improve the success of assisted reproduction techniques, the lack of further clinical trials is still present.

### Sperm DNA integrity

Assessing sperm DNA integrity is of great relevance to male infertility, as damage to sperm DNA can have a detrimental effect on fertilization, pre-implantation embryonic development and implantation (Ganeva *et al.*, 2021). Therefore, sperm DNA fragmentation testing has been used to achieve more in-depth knowledge about sperm quality due to the critical role of sperm DNA integrity for healthy embryonic development and successful reproductive outcomes (Esteves *et al.*, 2021).

Two articles that addressed the use of artificial intelligence to predict the integrity of sperm DNA were selected for this review. Noy *et al.* (2023) conducted a study to predict sperm DNA fragmentation using multilayer dye-free image data, including quantitative phase images and lightweight deep learning architectures using a prediction model that is based on the MobileNet convolutional neural network architecture. The results showed that the mean absolute error for cells with high prediction confidence is 0.05 and the mean absolute error of the 90th percentile is 0.1, where the range of the DNA fragmentation score is [0.1]. The study by McCallum *et al.* (2019) used a deep CNN trained to predict sperm quality. To train the neural network, they used an internal set of 1064 images of individual sperm cells of known DNA integrity. The results demonstrate not only the correlation between a cell image and DNA integrity (with bivariate correlation ~0.43), but also the model’s ability to distinguish cells of higher DNA integrity from the median with statistical significance. The trained model can evaluate an input sperm image and provide a DNA integrity prediction in less than 10 ms.

These studies are in line with that of Wang *et al.* (2019) who used sperm images from the original dataset to train machine learning algorithms, which demonstrated predictive ability regarding sperm DNA fragmentation (*r=*0.558 and 0.620 for linear and non-linear regression, respectively). The researchers found that there is a correlation between established morphological parameters and DNA integrity, which presents opportunities for better clinical sperm selection with the aid of machine learning. Dimitriadis *et al.* (2019) compared the conventional method of analysis with smartphone-based automated assessment of sperm DNA fragmentation and obtained preliminary results that demonstrated that a portable and inexpensive smartphone-based system can accurately measure not only the basic parameters of semen analysis, but also sperm functionality. Despite the great advances, more research is needed that addresses the use of artificial intelligence in assessing the integrity of sperm DNA.

### Sperm motility

Motility is an important parameter in ICSI, as it helps the embryologist to choose viable sperm for microinjection. In addition to poor fertilization due to the injection of non-viable sperm into the oocyte, the lack of motility in the sample can have an indirect negative effect on the fertilization result due to the delay in completing the microinjection procedure (Dcunha *et al.*, 2022).

Only one study relating the use of artificial intelligence in sperm selection to motility was found. Haugen *et al.* (2023) evaluated the performance of DCNN ResNet-50 in predicting the proportion of sperm in the WHO motility categories. Manual and DCNN-predicted motility was compared using Pearson’s correlation coefficient and difference plots. The strongest correlation between manually assessed mean values and DCNN-predicted motility was observed for % progressively motile sperm (Pearson’s r=0.88, *p*<0.001) and % immobile sperm (r=0.89, *p*<0.001). For rapid progressive motility, the correlation was moderate (Pearson’s r=0.673, *p*<0.001). The median difference between manual and predicted progressive motility was 0 and 2 for immobile sperm. The greatest bias was observed in high and low percentages of progressive 6 and immobile sperm. The value predicted by the DCNN was within the range of the inter-laboratory variation of the results for most of the samples. In line with the literature, this result aligns with the study by Goodson *et al.* (2017), where a web-based program, CASAnova, was developed to individually classify sperm into 5 different motility categories - progressive, intermediate, hyperactivated, non-vigorous and weakly motile - with 89.9% overall accuracy. Somasundaram & Nirmala (2021) trained an algorithm based on tail-head movement that is explained for motility analysis. This method provides the best sperm detection and identifies the sperm with the best motility in the group in a minimum run time of 1.12 s. Overall, the performance of the proposed algorithm has the capacity to be implemented to treat infertility, and these artificial intelligence models are capable of improving such automated tools, significantly increasing the accuracy, agility and reliability of assisted reproduction techniques, especially intracytoplasmic sperm injection (ICSI).

## CONCLUSION

The integration of AI represents a significant advance in assisted reproduction, and it is a great ally for fertility clinics. Through its ability to analyze large volumes of information, especially images and machine learning algorithms, AI can evaluate parameters such as morphology, motility and sperm DNA integrity. Thus, AI has proven to be satisfactory in sperm selection for ICSI, being effective in optimizing the embryologist’s time and minimizing variability between operators. However, further studies are needed to assess whether AI can improve fertilization rates, embryonic development and live births.
